# Cardiac Post-Chest Radiotherapy Complications in a 50-Year-Old Patient with Hodgkin Lymphoma

**DOI:** 10.3390/jcm12206506

**Published:** 2023-10-13

**Authors:** Aneta Klotzka, Karolina Sobańska, Sylwia Iwańczyk, Marek Grygier, Patrycja Woźniak, Maciej Błaszyk, Natalia Rozwadowska, Maciej Lesiak

**Affiliations:** 1 I Department of Cardiology, Poznan University of Medical Sciences, 61-848 Poznań, Poland; 2 Department of Radiology, Poznan University of Medical Sciences, 61-701 Poznań, Poland; 3 Institute of Human Genetics, Polish Academy of Sciences, 60-479 Poznań, Poland

**Keywords:** radiotherapy, pericarditis, pericardiectomy, Hodgkin lymphoma, valve degeneration

## Abstract

Lymphomas are a group of malignant tumors that originate in the lymphatic system. It is the most common type of blood cancer. It affects the lymph nodes, spleen, bone marrow, blood, and other organs. They can be aggressive or chronic. Hodgkin lymphoma survival rate is 2 in 100,000 people. Young adults aged 20–30 and people over 50 are most often affected. The prognosis of Hodgkin’s lymphoma is good, with a survival rate of up to 80 percent. Nevertheless, in 20–30 percent of patients who initially respond to treatment, the disease has a tendency to progress. The positive effect of radiotherapy (RT) on patients’ survival rates has been proven in many randomized clinical trials. Although the dose of chest RT has significantly reduced over the years, we still struggle with the long-term complications of post-RT repercussions, mainly because there is no established safe dose of RT affecting the heart. Other complications include earlier onset of coronary artery disease, early and late onset of pericarditis, valve degeneration (predominantly of the left heart), calcification of the aorta and its branches, heart failure, and arrhythmias. One patient can manifest each of the abovementioned complications, as in the present case. That is why choosing the right treatment strategy is crucial.

## 1. Introduction

Chest radiotherapy (RT) causes an increased risk of late complications, such as valvular heart disease, early onset of coronary artery disease, or pericardial disease. The effectiveness of oncological therapies often contributes to patients’ recovery or chronic disease status. Many patients repeatedly forget that they suffer from oncological disease and develop cardiac complications of RT. There are still discussions about the safe doses of radiation and which structures of the heart are most sensitive to the damage sustained from radiation therapy. More attention is also paid to minimizing the risk of possible complications of chest RT. The data show that the heart is an organ susceptible to damage during RT. Therefore, its exposure to radiation should be as low as possible. It is also recognized that we cannot yet unanimously establish a safe dose of RT. The 2022 European Society of Cardiology (ESC) Guidelines on cardio-oncology emphasize that the risk of radiotherapy-induced cardiovascular toxicity should be categorized based on the mean absorbed dose to the heart (mean heart dose, MHD), rather than the administered radiation dose, which may not accurately reflect the radiation exposure of the heart [1]. Nevertheless, the ESC guidelines emphasize that the MHD is not an ideal parameter because, in some patients, a very small heart area can sustain damage from a very high dose of radiation, which still poses a high risk despite the low MHD.

Most data on radiation complications in lymphoma are based on patients treated decades ago with the then-standard RT therapeutic dose of 40–44 Gy [2]. Currently, the doses used for tumors located in the chest are significantly lower. However, a significant linear correlation was found between the estimated MHD and the risk of cardiovascular disease. This risk increases by 1.5–7% per 1 Gy MHD. Many studies have also confirmed a significant relationship between the average doses affecting heart/myocardial structures and the long-term cumulative incidence of cardiac events, including coronary artery disease, heart failure, and valvular heart disease. 

Hematological malignancies include both children and young adults, among other groups of patients. Often, the exposure of the heart to radiation is combined with the concomitant administration of potentially cardiotoxic systemic therapy. Studies show that adverse cardiovascular events are the leading cause of non-cancer-related deaths in pediatric cancer survivors. Mulrooney et al. assessed >14,000 children treated between 1970 and 1986 in childhood cancer survivor studies. Researchers compared the incidence of heart disease in children with a history of cancer in a cohort of their siblings [3]. Thirty years after diagnosis, the incidence of heart failure, valvular heart disease, and pericardial disease was 3–4%. Over the years, the cumulative risk of cardiac complications after chest RT decreases. This decrease correlates with a reduction in doses of RT.

In addition to three-dimensional conformal RT, there are a number of options in modern radiation therapy to reduce the dose to the heart for breast and other cancers. These include abdominal positioning, breath management, breath-holding for deep inspiration and gating, intensity-modulated RT/volume-modulated arc therapy, and proton therapy. The beam pattern and beam energy are optimized for each individual anatomy [1].

In addition to the adverse effects of radiation itself, the patient’s cumulative risk of RT complications is also directly correlated with the underlying cardiovascular risk factors. Smoking, hypertension, diabetes mellitus, obesity (Body Mass Index, BMI > 30), age >65 years, and prior anthracycline therapy >250 mg/m^2^ are associated with future cardiovascular events in cancer survivors who receive chest radiotherapy. In patients with breast cancer undergoing RT, the prevalence of cardiovascular adverse effects increases the risk of major adverse cardiovascular events (MACE), whereas underlying ischemic heart disease is associated with a more than six-fold higher risk of MACE [4].

## 2. Case Report

**Figure 1:**
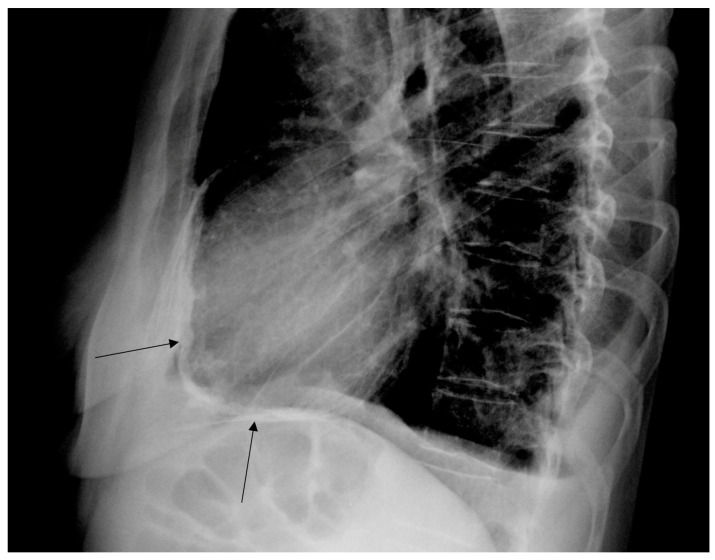
Constrictive pericarditis (arrows) (lateral chest X-ray).

**Figure 2:**
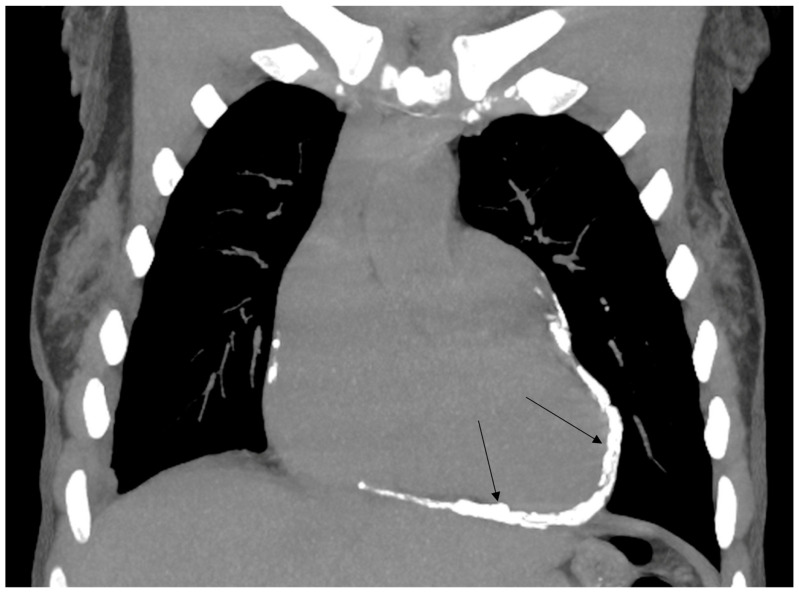
Constrictive pericarditis (arrows) (CT).

**Figure 3:**
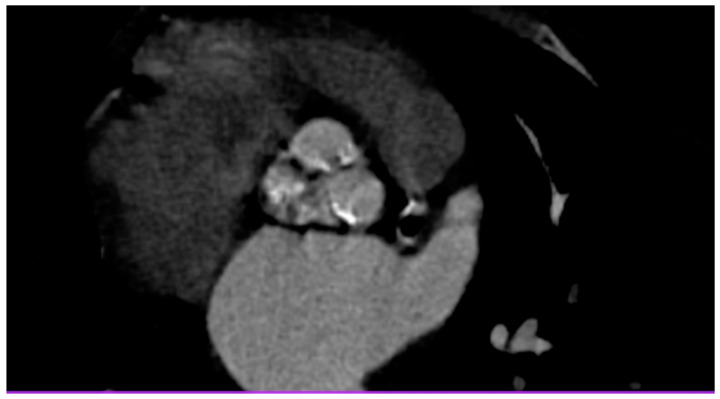
Calcified aortic valve (CT).

**Figure 4:**
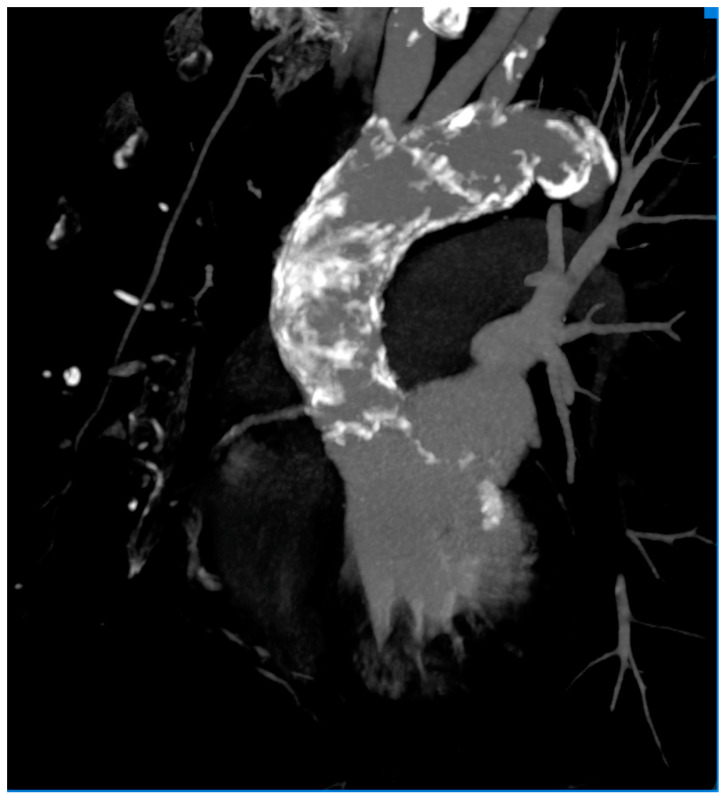
Porcelain aorta (CT).

**Figure 5:**
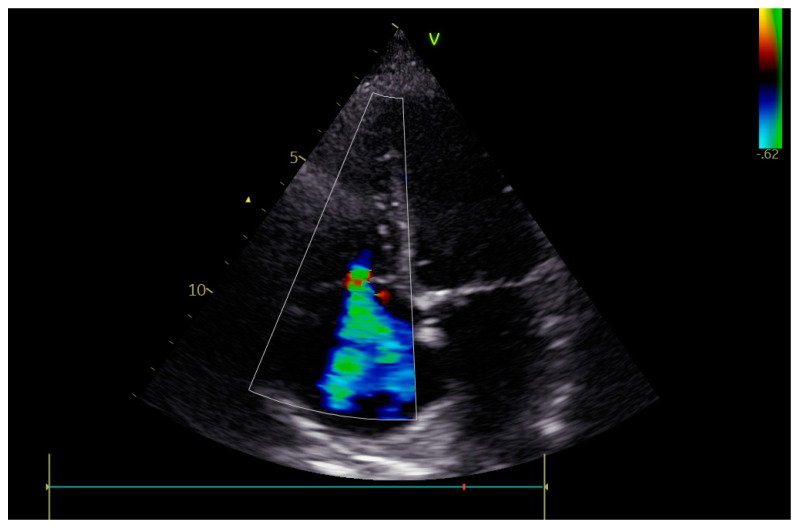
Residual tricuspid regurgitation (ECHO).

**Figure 6:**
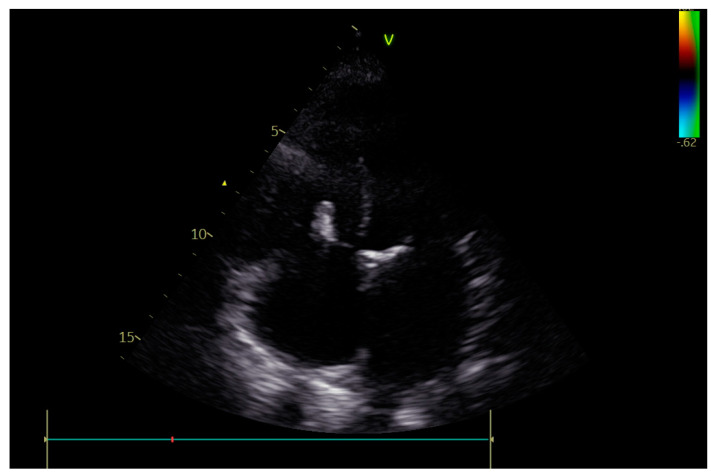
The TriClip implanted into the tricuspid valve.

A 50-year-old patient was admitted to the Department of Cardiology for percutaneous MitraClip implantation due to significant mitral regurgitation. The procedure was performed during hospitalization without any complications. MitraClip™ (Abbott Vascular, Santa Clara, CA, USA) is a simple procedure to fix the degenerated mitral valve. During the procedure, a small clip is implanted and attached to the mitral valve to improve the coaptation of the leaflets. The patient was treated 15 years ago for Hodgkin lymphoma. She received a chest RT dose of 35 Gy. Nine years after the completion of oncological treatment, the patient underwent cardiac surgery due to symptomatic constrictive pericarditis (Figure 1 and Figure 2). Even then, the aortic valve was moderately calcified (Figure 3). Fifteen years after the chest RT, the patient developed complex aortic valve disease with a predominance of significant aortic stenosis and significant tricuspid regurgitation. The CT scan also confirmed a porcelain aorta (Figure 4). We qualified our patient for the low invasive implementation of the biological valve in order to reduce the exposure to antithrombotic treatment since she had a higher risk of RT complications such as anemia, thrombocytopenia, and chronic renal failure. The patient had an aortic valve prosthesis implanted percutaneously (transcatheter aortic valve implantation, TAVI method). Six months later, the residual tricuspid regurgitation still required diuretic treatment (Figure 5). During the latest hospitalization, we implanted a TriClip device into the tricuspid valve (Figure 6). After two months, a follow-up echocardiographic examination revealed mild tricuspid regurgitation and a significant improvement in the patient’s general condition.

## 3. Discussion

Unfortunately, the complications of radiotherapy include not only the aortic valve but also the ascending aorta and its branches in a significant percentage. Data show that 60% of patients develop significant atherosclerosis of the ascending aorta after chest RT, and as many as 15% develop a porcelain aorta [5].

### 3.1. Pericarditis

Pericardial effusion is considered the most common late toxic adverse effect of chest RT [6]. The radiation-induced pericardial disease occurs when a significant area of the heart (>30%) receives a dose of 5000 rads. Although acute pericarditis after RT tends to be self-limiting, some patients develop chronic pericarditis. The chronic course of the disease affects 20% of Hodgkin lymphoma patients after the irradiation of the pericardium. As a result, the pericardial fibers thicken, become fibrotic, and often calcify [7]. This can lead to symptoms of pericarditis constrictiva, as observed in our patient nine years after chest RT.

### 3.2. Coronary Artery Disease

Radiation therapy to the chest is associated with an increased risk of ischemic heart disease. Radiation dosimetry research has shown that the left anterior descending artery (LAD) receives the highest radiation dose during RT for left-sided breast cancer (BC). This results in a higher incidence of LAD stenosis after RT in the left-sided BC compared to the right-sided BC. Heart radiation volumes and doses to the heart have decreased with the development of new techniques, although RT doses affecting the anterior wall of the heart still remain high. RT techniques using active control of breathing have shown a further reduction in radiation doses to the heart and LAD. The study showed a significant correlation between the simulated doses in LAD and subsequent stenosis in this artery. A study by Taylor et al. aimed to test whether there is a relationship between the radiation dose to the CA and subsequent coronary stenosis at this location, which requires coronary intervention. All patients received modern three-dimensional conformal radiation therapy (3DCRT), which provided patient-specific information on radiation doses for CA. The main focus was on the high-dose area of LAD. The study showed a significant correlation between the simulated doses received by LAD and subsequent stenosis in this artery [8].

Wennstig et al. also assessed the relationship between radiation doses to the coronary arteries and the location of the stenosis in the coronary vessels. The study population consisted of 182 women treated for breast cancer in Sweden from 1992 to 2012 [9]. Among the entire study group, 101 patients were treated for left breast cancer and 81 for right breast cancer. Women receiving mean doses of 1–5 Grays (Gy) to the mid-anterior descending branch of LAD had a significantly higher risk for subsequent coronary intervention compared to women receiving mean doses of 0–1 Gy to the mid-LAD. Women receiving mean doses of 5–20 Gy and above 20 Gy to the mid-LAD had an even higher risk of subsequent coronary interventions compared to women receiving mean doses of 0–1 Gy to the mid-LAD. The study proved that the radiation dose to LAD should be considered when planning RT treatment and that the dose should be as low as possible. Researchers suggest that minimizing the dose affecting LAD will reduce the risk of subsequent radiation-induced stenosis. Our patient had a coronary angiography performed before the pericardiectomy, and the coronary arteries showed no significant lesions.

### 3.3. Valvular Defects

Radiation therapy damages not only the valve leaflets but also the subvalvular apparatus. RT damage is usually not limited to a single valve because the area of tissue exposure to RT is larger. The causes for this phenomenon are seen in microdamage of the endothelium, progressive inflammation, and, as a result, fibrosis and calcification of tissues. Patients who received radiotherapy to the mediastinum had a 34-fold increased risk of valvular disease compared to the unexposed population in the Framingham study. Clinically significant valvular disease is found in 1% of patients after 10 years, 5% after 15 years, and 6% after 20 years of radiotherapy. The incidence of damage increases significantly after 20 years following the exposure to RT: mild aortic regurgitation has been reported in up to 45% of patients, moderate regurgitation in up to 15%, valvular stenosis of the left arterial orifice in up to 16%, mild mitral regurgitation in up to 48%, and mild pulmonary regurgitation in up to 12% of patients. Practically, 20 years after the completion of RT, a few patients have unaffected aortic valves [1]. 

Transthoracic echocardiography is the first-choice method to distinguish valve degeneration caused by rheumatic fever or senile degeneration from changes caused by RT. The lesions after RT are quite typical and include fibrosis and calcifications of the mitral and aortic valve leaflets located mainly in the basal and middle parts. Left heart valves are more frequently damaged. The involvement of the aorto-mitral curtain is also typical. The increase in the thickness of the aorto-mitral curtain correlates with an increase in perioperative complications of valve replacement. Significant valvular damage occurs significantly more frequently when the dose exceeds 30 Gy [10]. Doses of 20–30 Gy are considered safer for the heart, but most scientists believe that there is no safe dose of RT for the heart. In addition, previous exposure to chemotherapy treatment (mainly anthracyclines) may intensify degenerative changes in the valves. 

Our patient had significant aortic valve degeneration 15 years after RT with dominant aortic valve stenosis. Echocardiography also showed mild mitral regurgitation and significant tricuspid regurgitation. Our patient underwent cardiothoracic surgery when these significant heart defects were found, exactly six years following pericardiectomy due to constrictive pericarditis. 

In our patient, imaging tests also confirmed significant calcification of the ascending aorta. In such a situation, the key decision remains whether to perform the traditional open-heart aortic valve replacement or transcatheter TAVI (transcatheter aortic valve implantation) [10]. The calcification of the ascending aorta may preclude surgery by using the classical method. On the other hand, massive calcification of the aortic valve increases the risk of stroke. The 2017 ESC guidelines on the treatment of valvular heart disease indicate that in patients with risk factors, such as frailty syndrome, porcelain aorta, or a history of chest irradiation, the choice between surgical aortic valve replacement (SAVR) and TAVI should be made after a Heart Team consultation [11].

The latest ESC guidelines on cardio-oncology recommend TAVI as the method of choice in patients with intermediate operative risk and severe symptomatic aortic valve stenosis caused by chest RT [11].

In the case of our patient, the Heart Team decided to implant a valve using the TAVI method. If mitral regurgitation does not decrease after reducing the gradient by the aortic valve, we consider the percutaneous implantation of MitraClip and TriClip. Transcatheter valve repair and implantation techniques seem to be a safer alternative in this group of patients, especially considering the obligatory anticoagulation after traditional valve replacement, which increases the risk of bleeding [12]. This risk is even greater when combined antiplatelet and anticoagulant therapy is required. In such cases, it is recommended to shorten the period of antiplatelet therapy [13].

Finally, six months after TAVI, our patient still had symptoms of right ventricular heart failure, as tricuspid regurgitation was still significant. The patient was qualified for TriClip implantation. 

## 4. Conclusions

The morphology of the lesion after RT is not ideal for TriClip implantation, but the cusp coaptation sites are usually unaffected by calcifications, which allows the percutaneous procedure [14].

A separate issue is when significant symptomatic aortic stenosis is found at the time of the initiation of oncological therapy. Such patients should qualify for transcatheter TAVI treatment strategies. The transcatheter approach enables a faster improvement of patients’ condition and immediate continuation of oncological treatment. Patients undergoing oncological treatment have an increased risk of periprocedural complications, such as anemia, thrombocytopenia, worse kidney function, low protein levels, or hypercoagulability.

Oncological cancer patients with a higher risk of RT complications such as anemia, thrombocytopenia, worsening renal function, low protein levels, or hypercoagulability are a priori qualified for low invasive procedures or the implementation of biological valves so that they are not exposed to antithrombotic treatment.

## Data Availability

The data presented in this study are available on request from the corresponding author. The data are not publicly available due to privacy reasons.
